# Increasing resilience, sustainable village development and land use change in Tarumajaya village of Indonesia

**DOI:** 10.1038/s41598-024-82934-2

**Published:** 2024-12-30

**Authors:** Ida Widianingsih, Abdillah Abdillah, Djoko Hartoyo, Sapen Sartika Unyi Putri, Ahmad Zaini Miftah, Qinthara Mubarak Adikancana

**Affiliations:** 1 Public Administration Department, Faculty of Social and Political Sciences, Universitas Padjadjaran, Bandung, Indonesia; 2 Present Address: Graduate Program in Public Administration, Faculty of Social and Political Sciences, Universitas Padjadjaran, Bandung, Indonesia; 3 Center for Decentralization & Participatory Development Research, Faculty of Social and Political Sciences, Universitas Padjadjaran, Bandung, Indonesia; 4 Coordinating Ministry for Infrastucture and Regional Development, Jakarta, Indonesia; 5 International Relations Department, Faculty of Social and Political Sciences, Universitas Padjadjaran, Bandung, Indonesia

**Keywords:** Land Use Change, Inclusive development, Sustainable Village Development, Resilience and Sustainability communities, Environmental economics, Ecology, Ecology, Environmental social sciences, Natural hazards

## Abstract

This study explores land use change plans to be utilized fairly, sustainably, and resiliently for the benefit of rural community life in Tarumajaya Village, Indonesia. This study uses a qualitative method, with a case study approach to describe the land use change plan that can be utilized for the benefit of community livelihoods in Tarumajaya Village, Indonesia. This study indicates the need to increase access to land ownership for the Village government and local community. Based on field data, several alternatives can be pursued to expand the access of the community and village government to land, including utilizing part of PT. Lonsum Indofood Tbk’s land, PTPN VIII and Perhutani use change needs to be done to encourage resilient and sustainable village development innovation and realize justice and prosperity for the people of Tarumajaya Village, allocated for: (1) Agricultural Needs of the Rural Community; (2) Reserve Settlements; (3) Cemetery; (4) Health, Governance, and Education Facilities; and (5) Agricultural Areas and other productive ventures managed by the Village government. This study recommends a change of land use from PT. Lonsum Indofood Tbk, Perhutani to be managed by the Tarumajaya Village Government for the sake of sustainable and resilient village development, thus creating justice and prosperity for the community.

## Introduction

The issue of resolving global development problems outlined in the MDGs (Millennium Development Goals) agenda has not shown optimal results, thus the idea is continued by through the SDGs (Sustainable Development Goals) 2015–2030 agenda. SDGs aim to continuously improve the economic welfare of communities, maintain the sustainability of social life, preserve the quality of the environment, and ensure inclusive development and governance capable of maintaining the improvement of life quality from one generation to the next^1,2^. This agenda ensures that all countries are able to uphold global and national commitments in efforts to improve the welfare of society, covering 17 goals: (1) No Poverty; (2) Zero Hunger; (3) Good Health and Well-being; (4) Quality Education; (5) Gender Equality; (6) Clean Water and Sanitation; (7) Affordable and Clean Energy; (8) Decent Work and Economic Growth; (9) Industry, Innovation, and Infrastructure; (10) Reduced Inequality; (11) Sustainable Cities and Communities; (12) Responsible Consumption and Production; (13) Climate Action; (14) Life Below Water; (15) Life on Land; (16) Peace, Justice, and Strong Institutions; (17) Partnerships for the Goals^3–5^. These 17 SDGs are interconnected, emphasizing inclusivity, security, well-being, and sustainability as core issues for sustainable development in rural areas (urban, small towns, and villages)^6,7^.

The concept of sustainable development has been adopted by Indonesia in the National Medium-Term Development Plan (RPJMN) 2015–2019 document. Specifically, the SDGs agenda are translated into measurable programs, activities, and indicators as well as indications of financing support. In recent years, the strengthening of village autonomy has been reinforced by the implementation of Village SDGs regulated through the Minister of Villages Regulation No. 13 of 2020. The regulation prioritizes the use of village funds in 2021 to be directed towards efforts to achieve SDGs agendas. This Minister of Villages Regulation No. 13 of 2020 is based on thoughts related to the national development model based on Presidential Regulation Number 59 of 2017 regarding the implementation of sustainable national development goal achievements. Until now, two national evaluations of Village SDGs have been conducted in Indonesia. Based on the Ministry of Village and Disadvantaged Regions report, only 18 villages have met the SDGs 1 target: Poverty-Free Village, such as Sabdodadi Village-Bantul Regency, Daerah Istimewa Yogyakarta. In addition, 6 villages have met the SDGs 5 target: Rural Women’s Involvement, one of which is Petiken Village in Gresik, East Java. Furthermore, 100 villages have met the SDGs 7 target: Clean and Renewable Energy Village. One of them is Ringin Larik Village, Boyolali Regency, Central Java. Meanwhile, 10 villages have met SDGs 9: Village Infrastructure and Innovation according to Needs, such as East Bongkudai Village, East Bolaang Mongondow Regency, and North Sulawesi. Furthermore, 13 villages have met SDGs 17: Partnerships for Village Development, such as Canggu Village-Badung Regency, Bali^8^.

In this context, village SDGs essentially are policies that accelerate village development to improve the quality of life, livelihoods, and welfare of its communities^6,7^. The goals of village development according to Law Number 6 of 2014 concerning Villages are to improve the welfare of rural communities and the quality of human life as well as poverty alleviation through fulfilling basic needs, developing village facilities and infrastructure, developing local economic potentials, and utilizing natural resources and the environment sustainably. Sustainable rural development that is appropriate becomes a clear indicator socially, environmentally, and economically. Sustainable rural development for vulnerable groups in rural areas is intended to promote fair and sustainable development^6,7^. This sustainable village development is also inseparable from efforts to preserve the environment so that the provision of basic needs for the community such as clean water can continue to be met^9^. As stated by Mitchell^10^, one of the main challenges of sustainable rural development in Indonesia is inadequate water management and availability. Based on studies conducted by McIntyre-Mills et al.^4^ the issue of sustainable development has a similar pattern in various countries.

This study specifically discusses Tarumajaya Village as the research locus due to the following strategic factors: (1) Tarumajaya Village is located at KM 0 of the Citarum River; (2) The Citarum River flows through 12 administrative regions; (3) The Citarum River was once declared the most polluted river in the world in 2018^9^; (4) The governance of the Citarum River applies participatory governance (penta-helix); (5) Socio-economic and environmental problems of the Citarum River that need to be addressed urgently; and (6) The existence of the National Action Plan for the Citarum River based on Presidential Regulation No. 15 of 2018 concerning Acceleration of Pollution Control and Damage to the Citarum River Basin Area. Based on the results of the 2015 Bappenas study, the main problems occurring in Tarumajaya Village are land use change and the absence of agricultural land as a livelihood source for the community, resulting in a vicious circle of poverty and landlessness^4,11^.

At the macro level, the problem of Tarumajaya Village, Indonesia is also related to the inadequate policies related to watershed governance, thus sustainable watershed governance is needed. Sustainable watershed governance refers to the management and coordination of activities related to the sustainable protection, conservation, and use of water resources in a watershed area^12,13^. This involves collaboration and participation of various stakeholders, including government agencies, local communities, businesses, and non-governmental organizations, to ensure the long-term health and sustainability of watershed ecosystems^12^.

In line with the SDGs program, Bandung Regency carries out the Independent Village program^1^. Based on this program, which is concerned with environmental issues and is in line with the mission of Bandung Regency. The Bandung Regency government initiated the ‘*Kampung Bedas*’ or Prosperous Village Cleanup program, which is an integrated and participatory-based program that involves various sectors, utilizing local wisdom that makes a village the main actor in economic growth^14^. Villages are seen as the main actors in environmentally sustainable development because environmental problems are addressed there, not only environmental issues but also social and economic aspects^1,14^.This program aligns with the goals of sustainable development and management in Tarumajaya Village^11,15^.

The Management of Natural Resources with environmental culture and Rural Conservation Education Center tourism by Tarumajaya Village includes: (1) Encouraging Tarumajaya Village as a center for the development of rural conservation education tourism; (2) Village Development Priorities; (3) Encouraging the center of superior hard plant seedling agriculture; (4) Strengthening facilitation of multi-commodity agricultural system capital (Vegetables, Coffee, Avocado, fruits, etc.) through Village Owned Enterprises; (5) Protection of farmer rights in agricultural area management; (6) Provision of new residential areas for Village communities; (7) Arrangement of environmentally friendly livestock areas; (8) Equitable distribution and management of water resources for clean water for residents; (9) Waste management; (10) Providing land for the public cemetery of the Village community; 11. Development of rural conservation education tourism. In 2022, this village received the Best Tourism Village Award in the Pilot Category from the Bandung Regent^16–18^.

However, efforts to continue to improve village development and improve the welfare of the community in Tarumajaya Village, Indonesia are hindered by several problems, including land unavailability, slum settlements, and land dominance by industrial parties^15,19–21^. In addition, according to Village Information System from the Ministry of Village, the SDGs score of Tarumajaya Village in point number 2 (village without hunger) only reaches a score of 33.25 out of 100, point number 3 (healthy and prosperous village) only reaches a score of 39.10/100; point number 4 (quality village education) only reaches a score of 26.16/100; Village SDGs point number 8 (even economic growth of the village) only reaches a score of 24.53/100; even Village SDGs point number 9 (village infrastructure and innovation according to needs) only reaches a score of 6.05/100^22^. These data indicate that Tarumajaya Village still faces quite fundamental problems in achieving a prosperous and sustainable village. In addition, other actual problems in the field in Tarumajaya Village, Bandung Regency, West Java Province, where include an imbalance in land ownership between the community and the private sector (companies), which usually leads to land disputes or agrarian conflicts, which then lead to the loss of community living space and an increase in the number of poor people and this becomes the main problem in this village^11,15,19,21^.

The availability of land for the people in Tarumajaya Village, most of whom rely on agriculture for their livelihoods, is obstructed due to the lack of agricultural land. The limited land ownership in Tarumajaya Village, where the largest land ownership in the village is owned by plantation companies, namely PTPN VIII covering ± 1200 Ha (43.7%), PT. Lonsum Indofood, Tbk covering ± 627.4 Ha (22.9%), Perum Perhutani covering ± 819.9 Ha (29.9%), and only ± 97.7 Ha (3.6%) of land is owned by the community^19^. With a population of 15,386 people and a total of 4810 households, resulting in 500 households settling along the Citarum watershed and Perhutani Land, making the area a slum and very uninhabitable^1,11,15,19,21^.

Furthermore, the data shows that the number of residents occupying the Citarum riverbank is 236 households (Kp. Babakan Citarum RW 003). Meanwhile, the number of residents occupying poorly organized and slum settlements is 264 households (Kp. Ranca RW 022), 226 households (Kp. Citawa RW 023), and 181 households (Kp. Ranca RW 027). Meanwhile, residents occupying uninhabitable houses in the Perhutani land area are 218 households (Kp. Babakan Ranca RW 20), 217 households (Kp. Babakan Ranca RW 21), and 303 households (Kp. Pajaten RW 001)^11,15,19^. This data indicates the urgency of how land management oriented towards community welfare is crucial at this time. Therefore, with the above urgency, we believe that companies must contribute to supporting area arrangement programs made by the government.

The phenomenon of land use change has been studied by Prayitno et al.^23^ which shows that rice field land use has decreased by about 6.19% while residential land has increased by around 5.46%. This is caused by economic pressure. Although the government has issued policies related to Legal Protection of agricultural land, such as Law Number 41 of 2009 concerning Sustainable Food Agricultural Land Protection^24^. However, this has not been able to guarantee the protection of agricultural land area. Research by Suratha^25^ also supports this. The findings show that along with population growth and the development of human civilization, land ownership and use began to be disturbed. This disruption eventually creates complexity of problems due to population growth, discovery and use of technology, and development dynamics. Land that was originally used as a farming medium gradually changed into multifunctional use, and had an impact on food security in Indonesia^11,21,25^.

Land use change in Indonesia plays an important role in driving resilient and sustainable rural development. This is shown in various previous key studies on land transformation influenced by various factors, including government policies, community initiatives, and the integration of local knowledge into land management practices^6,7,21,26–29^. According to Annahar et al.^6,7^ and Ginting et al.^21^, the dynamics of land use change can enhance or weaken the resilience of rural communities, depending on how these changes are managed. In the study by Fedele et al.^27^, one important aspect of land use change is its impact on ecosystem services, which are critical to sustaining rural livelihoods. highlighted that rural communities in tropical landscapes, including Indonesia, have initiated local management practices to improve ecosystem services through diversified farming systems such as agroforestry, which combines cash crops with food crops. This approach not only improves food security but also strengthens the ecological resilience of the land, allowing communities to adapt to environmental changes and economic pressures. Furthermore, according to Afrizal & Berenschot^26^ the historical context of land use in Indonesia, characterized by crony capitalism and the priorities of economic elites, has often marginalized rural communities. This marginalization can lead to conflicts over land use, especially when corporate interests trump local needs. However, according to Aditya et al.^28^ initiatives that encourage participatory land management, can empower local communities by involving them in decision-making processes related to land use.

### Objective

The goal of this study is to investigate the effective and sustainable utilization of land use modification plans in Tarumajaya Village, Indonesia, in order to enhance the community’s sustainable livelihood while also ensuring the preservation of forest sustainability. The research divides itself into three primary areas: Initially, we aim to scrutinize the underlying causes of the challenges in changing land use during the expansion of Tarumajaya Village, Indonesia. The second objective is to analyze policy studies that promote land use change in order to foster sustainable and resilient development in Tarumajaya Village, Indonesia. This study aims to extract valuable insights from the transformation of land use in Tarumajaya Village, Indonesia, in order to promote sustainable and resilient development.

## Methods

### Research design

This study uses a qualitative method, with a case study approach^30,31^ to describe the land use change plan that can be utilized for the benefit of community livelihoods in Tarumajaya Village, Indonesia, fairly and sustainably while maintaining forest sustainability. The importance of qualitative research as a step/method in highlighting the types of problems to the important characteristics and research issues that are most appropriate for qualitative research in investigations to assess the quality of the meaning of research problems, causal flows, and developments of problems that require time (through specific approaches such as narrative, phenomenology, and up to case studies in qualitative research) cannot be overstated^30,31^. The use of qualitative methods through a case study approach in this study to explore, study, and identify various problems that cannot be easily measured about how land use change encourages sustainable development and resilience in Tarumajaya Village to provide significant benefits between community life, environmental sustainability, and sustainable village development, so that this study contributes to a complex and detailed understanding of the problems that occur in Tarumajaya Village.

Creswell & Poth^30^ defined case study research as a qualitative methodology in which researchers investigate a current bounded system (case) or several bounded systems (cases) over a period of time by collecting comprehensive and detailed data from various sources of information (such as observations, interviews, audiovisual materials, documents, and reports). They then report descriptions of the cases and identify the themes presented in them. In addition, Creswell & Poth^30^ proposed that the unit of analysis in a case study can consist of either several cases (multisite studies) or a single case (research conducted inside a single site).

Data sources include primary and secondary data obtained through literature research, public consultations/expert use, in-depth interviews, field observations, document studies, and literature review^32^. In this study, we examine the research data using a conceptual approach based on Elkington^33^’s Triple Bottom Line theory. This approach promotes a comprehensive perspective that considers social, economic, and environmental justice in the context of sustainable development.

### Data collections

The primary and secondary data, including field observations, interviews, government documents, literature review, online media, legislation, assessment findings, and other references, are analyzed using document survey methods^30,32^. The research data was collected based on Table 1.

**Table 1:** Data collations and data sources of the Primary Data.

Issues	Data Collations	Description	“Time is of the essence” and “Informant”
Existing conditions of Tarumajaya village	Field Observations	7 visits to Tarumajaya Village	1st : August 11–13, 2023 2nd : October 16, 2023 3rd : January 5–7, 2024 4th : January 17, 2024 5rd : February 6, 2024 6th : May 24–26, 2024 7th : August 20–21, 2024
Development Progress and Land Use Change in Traumajaya Village	In-depth interviews	10 key Informant Interview data, the primary data of this study, was collected through face-to-face, telephone, and paper interviews with 10 interviewees.	Head of Tarumajaya Village Secretary of Tarumajaya Village Chairman of Tarumajaya Bumdes Tarumajaya Village Consultative (*Badan Pembina Desa*,* BPD*) Body 7 Heads of Tarumajaya Hamlet Family Welfare Empowerment Movement (*Pemberdayaan dan Kesejahteraan Keluarga*, PKK) Tarumajaya Integrated Health Service Post (*Pos Pelayanan Terpadu*,* Posyandu*) Boarding House Owner Trader Leek Farmer Head of RW 09 Coconut Ice Trader
The problems, Challenges, and opportunities in land conversion towards a sustainable and resilient transformation of Tarumajaya village	Focus Group Discussion (FGD)	5 Times FGD in Tarumajaya Village	1st FGD (29 April 2023–30 April 2023) with (CDPD ALG Team (6 + 4 Research Assistant); Field Head; Geophysics KKN Team (7 Lecturer); Citarum Research Center Team (2 people)) 2nd FGD (16 October 2023) at the Tarumajaya Village Office regarding village innovation and participatory development. 3rd FGD (17 January 2024) at the Tarumajaya Village Hall about FGD and student observation progress reports to the apparatus and the community of Taruimajya Village. 4th FGD (6 February 2024) With (Prof. Ida ALG Team, Prof. Erna ALG Team, Asdep for Regional Development of the Ministry of Communications, Kaids PUTR Bandung Regency, Disbudpar, Kades, village apparatus, Tarumajaya Village community, KKN participants). 5th FGD (20 August 2024), With (Regent of Bandung: Dr. H. M. Dadang Supriatna, S.IP., M.Si. Head of Environment Division; Head of BPPD Member of the DPRD from the PKB faction Prokopim Bandung Regency Village Head: Mr. Ahmad Ihsan, S.E; Bumdes, Secretary, BPD, KIM of Tarumajaya Village; Youth Organization; Posyandu (28 cadres from 28 RW (Women)); Family Welfare Empowerment Movement (*Pemberdayaan dan Kesejahteraan Keluarga*, PKK); 15 cattle groups; Director of Kertasari Hospital; BPR Bank Representatives; Hamlet Heads; RW Chairmen; the Heads of Neighborhood (*Rukun Tetangga*, *RT*); Member of Mosque Management Committee (Alim ulama); Kertasari Sub-district Head; Police Chief; Farmer groups; Community Social Worker (*Pekerja Sosial Masyarakat*, PSM) 2 people (1 male and 1 female); Research Team of Fisip Unpad.

Appendix 1 of Law No. 12 of 2011 on the Formation of Law Regulations outlines normative legal procedures, which serve as a common normative juridical approach for analyzing secondary data derived from law, research findings, or other assessments. In addition, the data gathering process involves several methods, including organizing Focus Group Discussions (FGD) with key stakeholders in strategic development in Tarumajaya Village, conducting detailed interviews with influential village personalities, examining meeting records, previous research reports, and internal literature published by the Tarumajaya Village Government and Bandung Regency Government.

In addition, this study applies big data analysis to secondary data collection through online document surveys that specifically include the term “Desa Tarumajaya Kertasari.” As of November 12, 2023, this survey identified a total of 122 scientific papers, 271 scientific documents, and 657 scientific studies stored in the Google Scholar databases. We subsequently analyze the content of these documents by gathering, skimming, screening, and visualizing them to provide accurate and comprehensive results.

### Data analysis

**Fig. 1:**
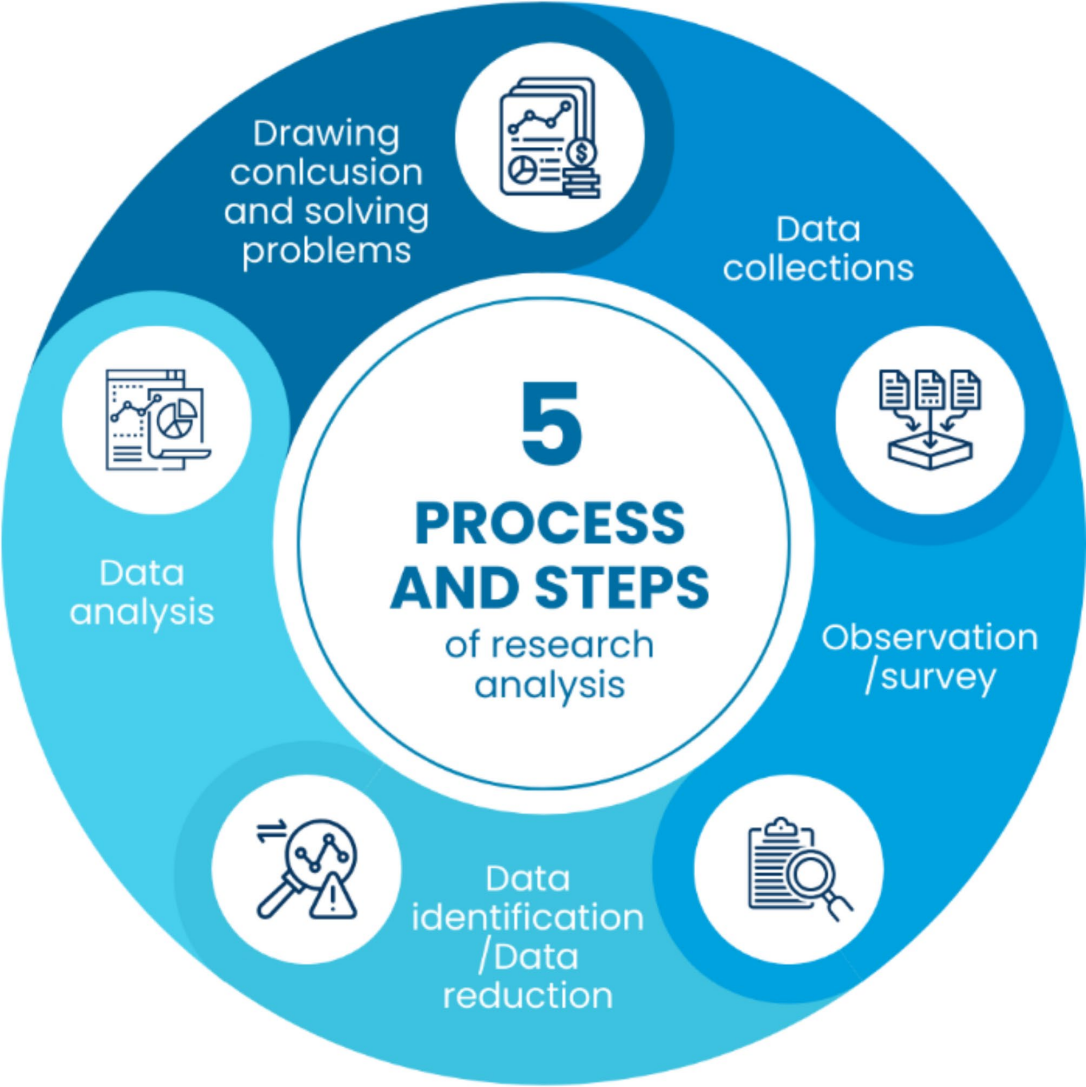
Process and steps of research analysis. Source: Processed from various sources, 2024.

The data, including regulatory, scientific, and empirical data, are examined utilizing interactive methodologies, as outlined by Miles et al.^34^, which has identified four distinct phases in the analysis process: data gathering, data reduction, data presentation, and conclusion formulation. Miles et al.^34^ identified data analysis as being encompassed within the final three processes. In legal research, this is implemented in the following manner: The process of data reduction encompasses the selection, simplification, and abstraction of data according to predetermined themes. Data display refers to the interpretation of data, where elements or totalities are given meaning and the results of data reduction are presented in narrative and/or tabular form in relation to the raised issues. Conclusion drawing and verification, the final stage of analysis, involve drawing conclusions to address the raised questions. This process is continuously verified throughout the research process to ensure the validity of the conclusions. The implementation of this procedure is illustrated in Fig. 1.

Based on Fig. 1, data analysis in this study is assisted by the Nvivo 12 Plus tool^35^ to explore, map, and draw various facts and findings in the research to derive the best conclusions.

### Research site

We conducted this study in Tarumajaya Village, Kertasari, Bandung Regency, in the West Java Province of Indonesia. Tarumajaya Village encompasses a variety of opportunities for ecotourism development within the framework of inclusive sustainable development. Tarumajaya Village is located at the base of Mount Wayang, at the Citarum River’s zero-kilometer bend in the Kertasari Sub-district of Bandung Regency. It is home to many popular tourist attractions, such as Situ Cisanti Zero Kilometer Citarum River, Bukit Paesan, Pine Forest Pakawa, Tawides, or Village Education Tourism Park. Furthermore, apart from these locations, there are also places dedicated to heritage and cultural tourism^16^. The Bandung Regency classifies Tarumajaya Village as one of the 50 emerging tourism communities. Situated at the base of Mount Wayang, at an elevation ranging from 1,600 to 1,920 m above sea level, the village embraces the concept of rural conservation education and tourism. It focuses on promoting educational principles, advocating for nature preservation, practicing environmentally sustainable agriculture and livestock farming, and fostering the growth of local arts and culture^11,16,18^.

**Fig. 2:**
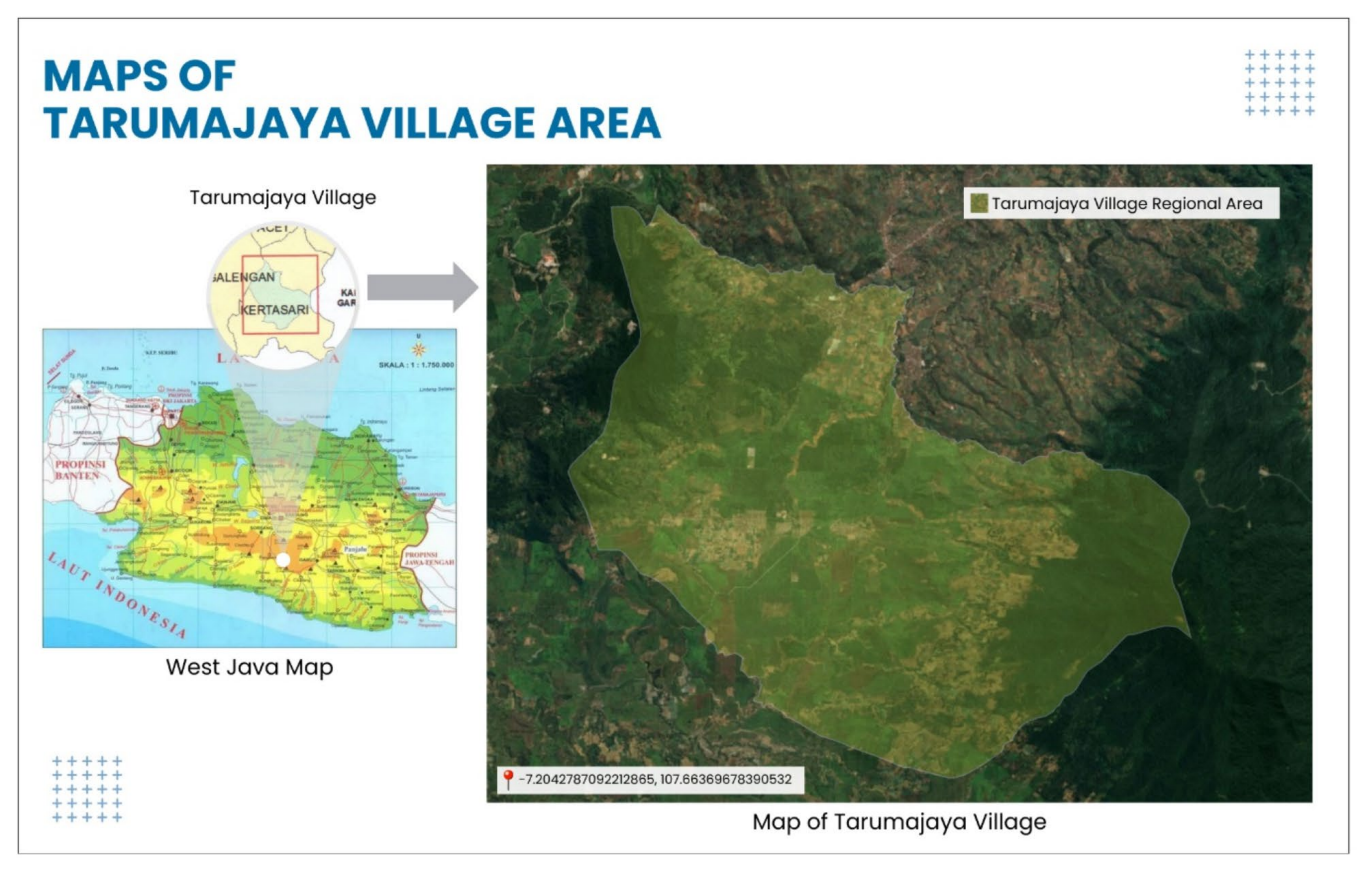
Map of Tarumajaya Village Area, Bandung Regency, West Java. Source: Processed by Google Earth (ArcGIS/GIS Software and Google Eart Pro Software), 2024.

Figure 2 illustrates that farming and agricultural labor are the primary means of subsistence for the residents of Tarumajaya Village. However, in addition to these capacities, there are several other prospects for developing Tarumajaya Village as a tourist attraction. These include: (1) Situ Cisanti, located 0 km away from the Citarum River Headwaters; (2) Hot Springs/Baths; (3) Curug Lodaya Kolot; (4) Tea Plantations; (5) Profusion of vegetables resulting from fertile agricultural land; (6) Potential for agro-tourism; (7) Capable of serving as afforestation areas with hard plant cultivation; (8) Strong sense of mutual collaboration and commitment to religious principles; (9) Possess cultural values that can be enhanced^11,19^.

## Results and discussion

### Roots of regional problems, land use change, and development of Tarumajaya village: an overview

One consequence of deficiencies in natural resource governance is the existence of land ownership imbalances, which in turn leads to land ownership polarization^36^. The land ownership problem is a significant concern in Tarumajaya Village. For agricultural nations such as Indonesia, land concerns are of utmost importance. Throughout history, conflicts have arisen due to the growing ownership of valuable lands by small landowners who often have political influence or wealthy individuals who utilize the natural resources of villages exclusively for their industrial growth (see Ayu & Heriawanto^24^, Givari & Ryski^20^).

This exploitation is in direct opposition to the provisions of the 1945 Constitution concerning land, nature, and wealth, which are designed to promote the well-being of the entire society. In conjunction with the introduction of capitalism in communities that rely on agriculture, where land is an essential requirement, the capitalist system has changed social interactions and power dynamics that either facilitate or impede the advantages of using agricultural resources, namely land^37^. Within a capitalist framework, land is a prime objective for investors seeking to optimize their financial gains^38^. Land has grown to be closely associated with wealth, while wealth is closely associated with influence. Increased land ownership results in greater financial resources and increased influence for the individual^38^.

Land, synonymous with power, drives competition for land ownership. Thus, when capitalism enters villages based on agriculture, agrarian capitalism shapes farmers from subsistence to reinvestment, accumulation, and business scale expansion through agricultural production^39^. Hence, subsistence commodification to fulfill these purposes has led these capitalist farmers into the market system. Subsistence commodification has resulted in many farmers who were once self-sufficient becoming increasingly dependent on the market to meet their daily and reproductive agricultural needs^40^. Consequently, they become more reliant on money. This situation benefits wealthy farmers who own capital as they accumulate wealth, one of their goals being to buy land to expand their business scale. Thus, the gap in land ownership widens^40^.

**Fig. 3:**
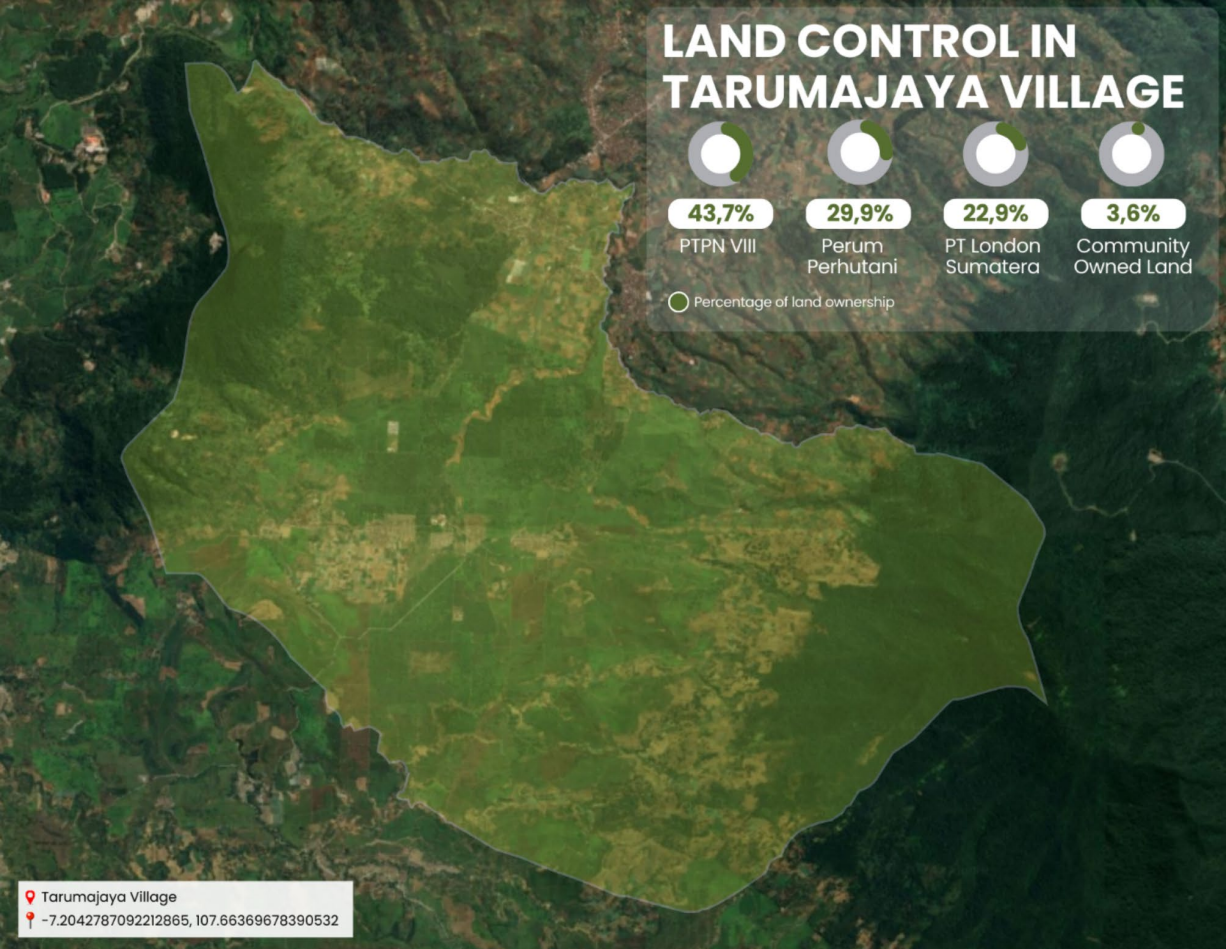
Percentage of Land Control in Tarumajaya Village, Bandung Regency, Indonesia. Source: Processed by Bandung Regency Government (using Google Eart Pro Software), 2024.

Based on Fig. 3. Tarumajaya Village document report (2023), Tarumajaya Village faces issues such as (1) land limitations; (2) Slum Settlements; (3) Poverty & Uninhabitable Houses; (4) Environmental Pollution; & Clean Water. For example, land limitations in Tarumajaya Village, where the largest land ownership is held by plantation companies, namely PTPN VIII covering ± 1200 Ha (43.7%), PT. Lonsum Indofood, tbk covering ± 627.4 Ha (22.9%), Perum Perhutani covering ± 819.9 Ha (29.9%), and only ± 97.7 Ha (3.6%) of land is owned by the community. With a population of 15,386 people and 4,810 households, approximately 500 households reside along the Citarum River Basin and Perhutani Land, making the area a slum and highly uninhabitable^11,19^. Regarding these issues, we believe that all companies must contribute to supporting area management programs initiated by the government.

**Fig. 4:**
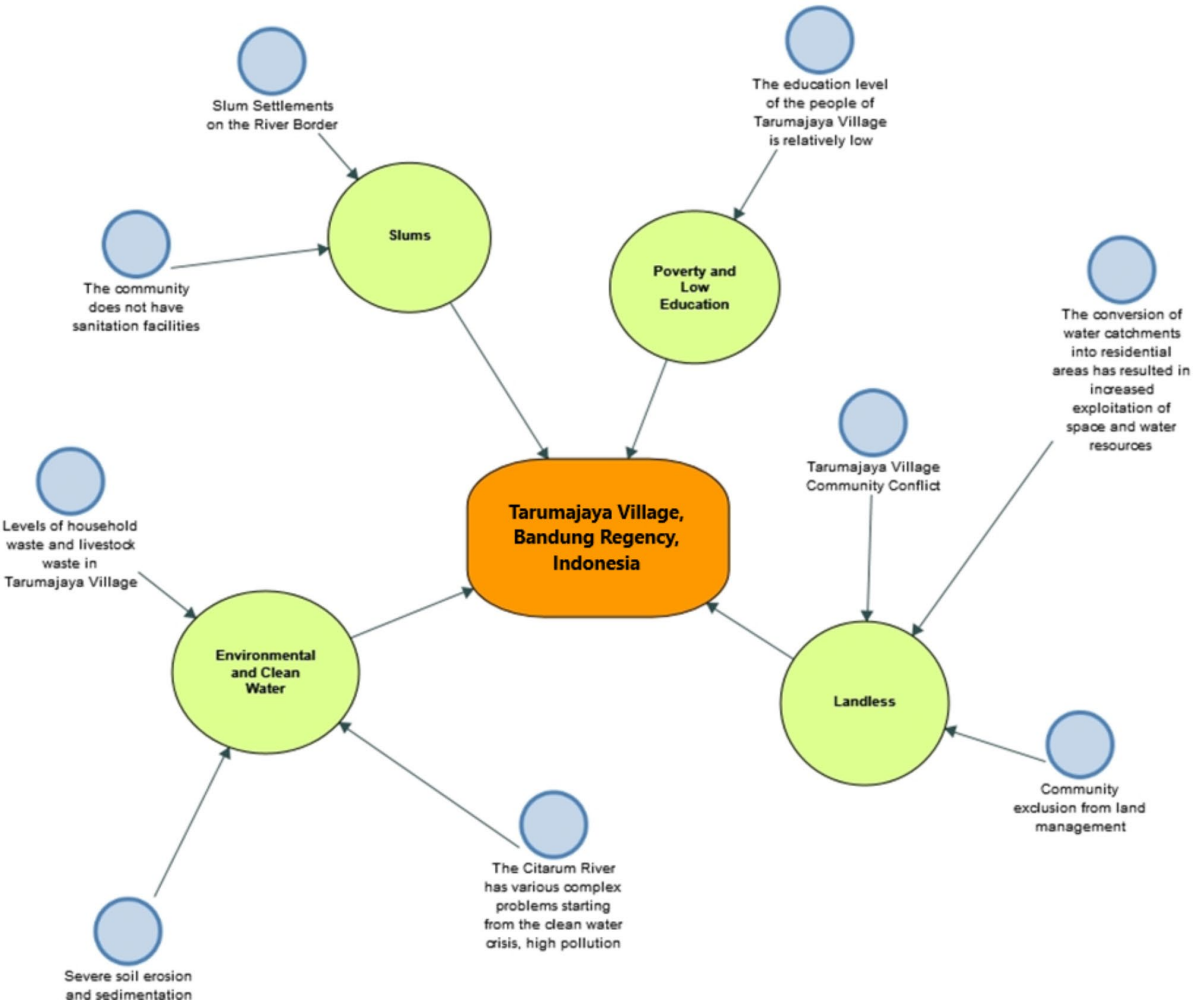
The Roots of Problems in Sustainable and Resilient Village Development in Tarumajaya Village, Indonesia. Source: Processed via Nvivo 12 Plus, 2024

In addition to the challenges faced by Tarumajaya Village (see Fig. 4), there are also opportunities in Tarumajaya Village, including Integration of Environmental Preservation Programs, Community Economic Development, and Area Management, Village Economic Improvement, Tarumajaya Sustainable Environmental Policy (Local Policy), Tarumajaya Village-Owned Enterprises, Tarumajaya Information and Communication Group as a community group excelling in IT, Forest Resource Management, Spiritualism, Community-based Ecotourism Development, Alternative Energy Use, New Business Strategies for Tarumajaya Village Tourism Business Units, Spatial Planning, Religious Communication Activities, Community Literacy Enhancement, Public Health Sector Improvement, Citarum Harum Environmental Policy Strategies, Online Monitoring (Onlimo) System-Based River Pollution Control, Efforts to Achieve Increased Citarum River Water Quality Index Values, Household Waste Utilization, Citarum River Restoration with Military Involvement, Collaborative Governance Program for Citarum Harum in Improving Citarum River Water Quality, Citarum Hulu Watershed Flood Mitigation Based on the Hec-Hms Model, Development of Skills and Detection of Income Sources for Lakupandai Partnerships with Banking, Environmentally Based Local Wisdom Communication in the Citarum Watershed, Supporting Strategies for Education, Literacy, and Financial Inclusion, and in the field of Agroforestry.

Furthermore, another actual problem in Tarumajaya Village, Bandung Regency, West Java Province, where there is an imbalance in land ownership between the community and the private sector (companies), which usually leads to land disputes or agrarian conflicts, resulting in the loss of community living space and an increase in the number of poor people, and this is the main issue in this village^11,15,19,41^.

### Policy study and land use change in Tarumajaya village: towards resilient and sustainable rural development and development

The application for land use from PT Lonsum Indofood Tbk, Perhutani, dan PTPN VIII by Tarumajaya Village aims to improve the community’s and village’s access to land use, to formulate management plans, and to regulate its implementation so that the utilization of village land can benefit the village community’s livelihood fairly and sustainably, while also preserving the forest, promoting innovation, and fostering the development of Tarumajaya Village^11,15,19,41^.

The purpose and intention regarding the need for land from PT Lonsum Indofood Tbk, Perhutani, dan PTPN VIII by for the needs of the forest, community agricultural gardens, public and social facilities, health facilities, educational facilities, burial facilities, and the village government in Tarumajaya Village are based on policies^11,15,19,41^. Berikut berbagai perubahan lahan untuk pembangunan yang lebih berkelanjutan dan berketahanan di Desa Tarumajaya (see Table 2).

**Table 2:** Land use change to sustainable and resilience Tarumajaya Village Development.

Land Owner	Village Land Transformation	Allocation and Progress
PT Lonsum Indofood Tbk	A proposal to acquire 40 Ha of PT Lonsum Indofood Tbk land for Tarumajaya Village Government	Expansion of tourism (10 Ha) Educational mand sports Facilities, Pilardua Settlement (10.5 H) Bedas Village (10) Cemetery Facilities (1 Ha) Government office and service Facilities (0.5 Ha) Educational Facilities/State Vocational Schools (1) Barn/Village Productive Asset.
Perhutani	The village government propose 962 ha of the perhutani’s land to be managed by the Village forest management institution (*Lembaga Pengelola Hutan Desa*, LPHD) Jaya Nirwana The village government plans to allocate the land for general and Social facilities managed by Tarumajaya Village	Some of the Perhutani forest located in Tarumajaya Village that covers an area of 962 Ha. Residential areas (10 Ha) Public Facilities (fasum)/Social Facilities (Fasos) (10.5 Ha)
*Resolution of Land Ownership in Forest Areas* *(Penyelesaian Penguasaan Tanah dalam Kawasan Hutan*,* PPTKH)*	Provision of 8.1 Ha of land to the Tarumajaya Village Government	945 houses (settlements) 15 public facilities
Tarumajaya Village	Of the entire area that will be managed by the village, namely the total land area: 40 Ha, 926 Ha, and 81 Ha.	Village and community designation. Public facilities and social facilities (to promote village sustainability and resilience)

Source: Processed by various references, 2024.

This application is supported by various policies such as Article 33 paragraph (3) of the 1945 Constitution, which states, “the land and water and the natural riches contained therein shall be controlled by the state and used for the greatest prosperity of the people.” In addition, the Regulation of the Minister of Agrarian Affairs and Spatial Planning/Head of the National Land Agency of the Republic of Indonesia Number 18 of 2021 Regarding the Procedures for Determining the Management Rights and Rights Over Land in Article 82 paragraph 1 which states if the Applicant is a legal entity in the form of a limited liability company including state-owned enterprises/regional-owned enterprises and its use is for plantations, it is required to facilitate the development of community gardens around 20% (20%) of the land area applied for by the Right to Use Business for the surrounding community.”

Regulation of the Minister of Agrarian Affairs and Spatial Planning/Head of the National Land Agency of the Republic of Indonesia Number 18 of 2021 Regarding the Procedures for Determining the Management Rights and Rights Over Land in Article 73 paragraph 1 regarding the Requirements for Extension and/or Renewal of the Right to Use Business originating from State Land letter I number 8 which states “willing to release land for public purposes either in part or in whole.” Based on Government Regulation No. 23 of 2021 concerning Forestry Management, this Policy includes the need for social forestry in villages as community forests or community plantations managed by village governments intended to improve the quality of life of the community, increase welfare, environmental balance, and social-cultural development. Based on Presidential Regulation of the Republic of Indonesia Number 86 of 2018 concerning Agrarian Reform, land obtained from the obligation of the HGU holder to surrender at least 20% (20%) of the land area of the HGU that has changed to HGB due to changes in spatial planning (see Article 7). Also, according to the Regulation of the Minister of Public Works and Public Housing of the Republic of Indonesia Number 28/Prt/M/2015 concerning the Determination of River Border Lines and Lake Border Lines which stipulates the rules for small river border lines without embankments outside urban areas as referred to in paragraph (1) letter b, determined at least 50 (fifty) meters from the left and right edges of the river bed along the river channel (see Article 6 paragraph 3).

Based on the above, in relation to the expiration of the business use rights of PT. Lonsum Indofood’s in December 2023, the Head of Tarumajaya Village submitted a land application based on the aforementioned reasonings. For the mechanism, the village government is ready to follow and implement in accordance with the Regulation of the Minister of Agrarian Affairs and Spatial Planning/Head of the National Land Agency of the Republic of Indonesia Number 18 of 2021 concerning the Procedures for Determining the Management and Rights Over Land. This is in line with the goal of protecting and empowering farmers in Bandung Regency Regulation No. 10 of 2021 to achieve the welfare of farmers as the main actors in achieving the success of agricultural development and contributing to the sustainability of self-sufficiency, sovereignty, and food security, protection and empowerment are needed. Farmers in Tarumajaya Village need access to agricultural land where limited land ownership by the community in Tarumajaya is one factor in increasing the vulnerability of the community there^11,19,41^.

## Discussions

The acceleration of development innovation in Tarumajaya Village is hindered by the limited land. This is not in line with the concept of a resilient and sustainable village, where sustainable development towards a just village in its principles and practices enhances the quality of life of the community as a whole^11,19,41^. The focus of even development in rural areas, through the use of technology and innovation, collaboration of each stakeholder, and encouraging sustainable development to improve the quality of life and welfare of the community^11,19^. Thus, land requests for various village purposes as mentioned earlier can be utilized and managed by the Tarumajaya Village government, which is expected to receive support from the Bandung Regency government^11,19,41^. Law No. 23 of 2014 on Regional Governments stipulates that the implementation of regional governments such as the Bandung Regency Government needs to be improved by paying more attention to the aspects of governance from the national to the village level, thus the regency government will provide aid to Tarumajaya. Also, Law No. 6 of 2014 concerning Villages which states that rural areas are areas that have main activities in agriculture, natural resource management, settlement management, social services, economic activities, and village governments that carry out development. In the context of national laws, Regulation of the Minister of Environment and Forestry of the Republic of Indonesia No. 4 of 2023 stipulates that villages manage village forest areas to be used for village welfare and empowering the community, as well as for forest resource conservation.

This study finds the importance of understanding the root of problems at the community level, the principles of sustainable development models that strive to balance social, economic, and environmental aspects as proposed by Elkington (Triple Bottom Line) need to be continuously encouraged and adapted in village development governance. One key aspect of sustainable and resilient village development, carried out through empowering local communities to actively participate in the development process and increasing the capacity of village governments in managing village development.

Furthermore, this study emphasizes that achieving sustainable development and active community participation in development can only happen with support from various parties and having access to resources (such as land). In this regard, creating space and facilitating access to land use for communities and village governments can be a solution to achieving sustainable and resilient village development.

It should be understood that land use change should be utilized and managed by the Tarumajaya Village government for the greatest benefit of the community. The expiration of the business use rights of PT. Lonsum Indofood Tbk’s HGU contract may be the right solution to provide opportunities for the Tarumajaya Village community and village government to obtain access to land, which has been very limited. This effort needs support from the Bandung Regency government, the Indonesian House of Representatives, the Bandung Regency Regional People’s Representative Council, the National Land Agency, as well as other strategic actors.

As for the mechanism for implementing land use conversion, it can be carried out in accordance with the Regulation of the Minister of Agrarian Affairs and Spatial Planning/Head of the National Land Agency of the Republic of Indonesia Number 18 of 2021 concerning the Procedures for Determining the Management and Rights Over Land. Land use conversion needs to be carried out to encourage innovative and sustainable village development and to realize justice and welfare for the Tarumajaya Village community, allocated for: (1) the needs of the village community’s agriculture; (2) Residential reserves; (3) Burial grounds; (4) Health, government, and education facilities; and (5) Agricultural areas and other productive activities managed by the village government. This study recommends the conversion and use of part of the land (Total land application 196 Ha or 20% of HGU) from PT. Lonsum Indofood Tbk to be managed by the Tarumajaya Village government for the purposes of sustainable and resilient village development, thus creating justice and welfare for the community^11,19,41^.

To realize sustainable and resilient villages in Tarumajaya Village, it is necessary to adjust to the duration of the business use rights of PT Lonsum Indofood Tbk’s contract. For example, if a contract extension is given for 25 years, then the Village Government with the support of the Regional Government and other parties needs to prepare land governance and utilization for the next 25 years, until the business use rights PT. Lonsum Indofood Tbk’s is next extended, especially for the needs of the Tarumajaya Village community and government. Considering the position of Tarumajaya Village located at KM 0 Citarum, this area needs to be supported to become a conservation village that applies sustainable and resilient development models. One strategy in achieving this goal can be implemented by creating a pilot project for the Smart Integrated Farming System area that fits the conditions of Tarumajaya Village^11,19^. With quality village human resources, especially the young generation of KIM (Community Information Group) activists who became National champions in 2023 in Surabaya, the idea of a Community-based Smart Integrated Sustainable Farming System Development Model in Tarumajaya Village can be realized. This development model is recommended in land use and sustainable village development in Tarumajaya Village, which is an integrated and intelligent agricultural system based on community involvement^11,19^. The approach used refers to local potentials (agriculture, animal husbandry, plantations) which progress by adopting technological advances, encouraging community involvement, and implementing sustainable practices^11,19^. This model is believed to be able to encourage efficient and environmentally friendly food production. This system aims to overcome various challenges in agriculture, such as climate change, food insecurity, and inequality^42–46^. The Bandung Regency Government, the Indonesian House of Representatives, the Bandung Regency Regional People’s Representative Council, the National Land Agency, as well as other strategic actors are expected to encourage the redistribution of assets (equal distribution of land ownership for the needs of the community and village government in fulfilling various primary and secondary needs in Tarumajaya Village). This is related to the goals of SDGs in villages and the realization of independent villages in Bandung Regency, Indonesia.

### Research limitations

The limitation of this study is that it only highlights Land Use Change in Tarumajaya Village, Bandung Regency, Indonesia, and land use conversion issues also occur in various villages in Indonesia. However, this study can be a lesson that can be used to overcome land use change issues that are widespread in various villages in Indonesia in promoting village sustainability and resilience. To improve this study, future researchers are advised to examine land use change issues in promoting sustainable and resilient development in various villages facing similar issues in Indonesia.

## Conclusions

This study’s summary examines the intricacies of land use change in Tarumajaya Village, highlighting the interaction of local governance, community requirements, and sustainable development. It underscores the critical problem of restricted land access, which obstructs the village’s development and innovation. The report advocates for a transformative approach to land management, proposing the reallocation of land currently owned by large enterprises like PT Lonsum Indofood Tbk to meet the community’s agricultural, residential, and social needs. The research emphasizes the significance of collaborative efforts in attaining equitable land distribution by promoting active involvement from local inhabitants and strengthening village government capabilities. Furthermore, it emphasizes the importance of harmonizing land use policies with sustainable development principles, ensuring that environmental preservation and community well-being are prioritized. The topic necessitates a collaborative endeavor among diverse stakeholders, including governmental agencies and private organizations, to establish a framework that fosters resilient and inclusive village development, thereby tackling the historical issues encountered by Tarumajaya Village, Indonesia.

This study concludes that the village government and local community must do more to increase their access to land ownership. According to field data, there are several ways to expand community and village government access to land, one of which is by utilizing part of the land of PT Lonsum Indofood Tbk, Perhutani, dan PTPN VIII. The land must be converted and used to encourage innovative and sustainable village development and to achieve justice and welfare for the Tarumajaya Village community. The land must be allocated for: (1) the needs of the village community’s agriculture; (2) Residential reserves; (3) Burial grounds; (4) Health, government, and education facilities; and (5) Agricultural areas and other productive activities managed by the village government. Tarumajaya Village, Indonesia, which is a location with a high level of vulnerability and the hindrance of development innovation acceleration attempted by the village government due to limited land. This study offers several alternative steps that can be synergized in addressing village development issues and land use conversion in Tarumajaya Village, Bandung Regency, Indonesia.

Land use change can be carried out by referring to several regulations, for example, the Regulation of the Minister of Agrarian Affairs and Spatial Planning/Head of the National Land Agency of the Republic of Indonesia Number 18 of 2021 concerning the Procedures for Determining the Management Rights and Rights Over Land in Article 73 paragraph 1 regarding the Requirements for Extension and/or Renewal of the Right to Use Business originating from State Land letter I number 8 which states “willing to release land for public purposes either in part or in whole”. Meeting the land needs for community agricultural gardens, public and social facilities including health, education, environmental facilities, burial grounds, and government needs to be encouraged in rural areas such as in Tarumajaya Village facing land limitations. This can encourage innovative village development and create justice and welfare as stated in the principles of SDGs. Land use change can meet the land needs for former PT Lonsum Indofood Tbk employee residential reserves for the next 25 years when the HGU is extended and the relocation of existing houses on the banks of the Citarum River, which is dominated by former employees of PT. Lonsum Indofood Tbk and PTPN VIII. So that in the future, there will be no more conflicts as has occurred in the past few years with the surrounding community (in 2013, 2019) or other institutions (in 2014, 2016) regarding boundaries or cultivation.

This study highlights the essential requirement for improved access to land ownership for the community and local government in Tarumajaya Village, asserting that sustainable development depends on equitable land use practices. The results indicate that the strategic use of land possessed by major organizations, such as PT Lonsum Indofood Tbk and Perhutani, can substantially enhance the village’s welfare. The study promotes a holistic land management strategy that reallocates property for vital community necessities, including agriculture, housing, healthcare, education, and burial sites, thereby prioritizing the welfare of the local populace. Moreover, incorporating community participation in decision-making is crucial to guarantee that development projects correspond with the desires and requirements of the population. This study underscores the issues encountered by Tarumajaya Village while providing pragmatic solutions to enhance resilience, advance environmental sustainability, and encourage equitable growth, thus laying the foundation for a more successful future for the community.

## Data Availability

The study results can be found in the figures attached to the article. The data set used to support the findings of this study is available from the corresponding author upon request.
